# A Unique Dual-Readout High-Throughput Screening Assay To Identify Antifungal Compounds with Aspergillus fumigatus

**DOI:** 10.1128/mSphere.00539-21

**Published:** 2021-08-18

**Authors:** Sarah R. Beattie, Damian J. Krysan

**Affiliations:** a Department of Pediatrics, Carver College of Medicine, University of Iowa, Iowa City, Iowa, USA; b Department of Microbiology and Immunology, Carver College of Medicine, University of Iowa, Iowa City, Iowa, USA; Hackensack Meridian Health Center for Discovery and Innovation

**Keywords:** *Aspergillus fumigatus*, adenylate kinase, antifungal drugs, cell lysis, filamentous fungi, high-throughput screening

## Abstract

Treatment of invasive mold infections is limited by the lack of adequate drug options that are effective against these fatal infections. High-throughput screening of molds using traditional antifungal assays of growth is problematic and has greatly limited our ability to identify new mold-active agents. Here, we present a high-throughput screening platform for use with Aspergillus fumigatus, the most common causative agent of invasive mold infections, for the discovery of novel mold-active antifungals. This assay detects cell lysis through the release of the cytosolic enzyme adenylate kinase and, thus, is not dependent on changes in biomass or metabolism to detect antifungal activity. The ability to specifically detect cell lysis is a unique aspect of this assay that allows identification of molecules that disrupt fungal cell integrity, such as cell wall-active molecules. We also found that germinating A. fumigatus conidia release low levels of adenylate kinase and that a reduction in this background allowed us to identify molecules that inhibit conidial germination, expanding the potential for discovery of novel antifungal compounds. Here, we describe the validation of this assay and proof-of-concept pilot screens that identified a novel antifungal compound, PIK-75, that disrupts cell wall integrity. This screening assay provides a novel platform for high-throughput screens with A. fumigatus for the identification of anti-mold drugs.

**IMPORTANCE** Fungal infections caused by molds have the highest mortality rates of human fungal infections. These devastating infections are hard to treat and available antifungal drugs are often not effective. Therefore, the identification of new antifungal drugs with mold activity is critical. Drug screening with molds is challenging and there are limited assays available to identify new antifungal compounds directly with these organisms. Here, we present an assay suitable for use for high-throughput screening with a common mold pathogen. This assay has exciting future potential for the identification of new drugs to treat these fatal infections.

## INTRODUCTION

In general, the therapeutic options for treating life-threatening fungal infections are much more limited than those available to treat almost any other type of infection. Currently, there are only three classes of clinical antifungals used as primary therapies to treat invasive fungal infections. These options become even more limited when treating patients with invasive mold infections (IMI) due to the acquired or intrinsic resistance of many molds to antifungals, drug-drug interactions, and dangerous drug toxicity for critically ill patients. Aspergillus fumigatus is the most common agent of IMI, causing infections called aspergillosis. Although mold-active azoles (e.g., voriconazole) and amphotericin B have potent *in vitro* activity against A. fumigatus (1), these drugs have significant drug-drug interactions and toxicity, respectively, and resistance to azoles is emerging in A. fumigatus clinical isolates (2, 3). Furthermore, treatment failure for aspergillosis is 40 to 70% (4) and mortality rates are ∼60%, but can be up to 90% depending on underlying disease (5). For the rarer molds, such as Fusarium spp., only amphotericin B has consistent *in vitro* activity against clinical isolates, while susceptibility to azoles is much more variable and echinocandins have no activity (6, 7). For many of the rarer molds, drug therapy has little proven clinical efficacy, leading to treatment failure and mortality rates of up to 100% (4, 8). Clearly, expanding our ability to treat medically important invasive mold infections represents an important unmet need in the field.

One possible reason for the limited activity of current antifungal drugs against molds is that antifungal drug screening has primarily focused on yeasts due to a larger perceived market for yeast-targeted drugs. Another reason is the technical difficulties of screening molds using standard growth-based assays. Hyphal cultures are heterogeneous, which, combined with growth at the air-liquid interface, reduces the correlation between optical density (OD) measurements and biomass increase. As a result, OD-based assays can only reliably detect molecules that completely inhibit the germination of conidia. As an alternative to optical density as a readout, growth-based assays using metabolic dyes, such as resazurin, have been used for high-throughput screening of A. fumigatus (9). While metabolic dye-based assays have improved sensitivity compared to optical density measurements, growth-based assays are still relatively limited in their sensitivity. More generally, expanding our repertoire of assays beyond traditional growth-based methods could avoid rediscovering the same classes of molecules over and over again (8).

**FIG 1:**
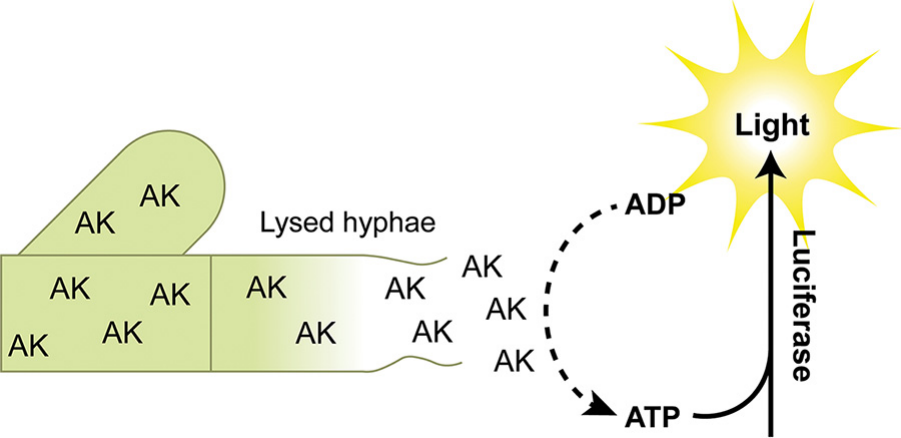
The adenylate kinase assay detects cell lysis. Release of the cytosolic enzyme adenylate kinase (AK) is detected using a reagent that contains ADP, which is converted to ATP by AK. The ATP drives the formation of light by the oxidation of luciferin by luciferase.

To fill the gap in non-growth-based assays for high-throughput screening with filamentous organisms, we developed an assay for anti-mold activity that uses the release of adenylate kinase (AK) into the growth medium as a readout of A. fumigatus cell lysis. As shown in Fig. 1, AK is a ubiquitous cytosolic enzyme that is released into the growth medium when a fungal cell compartment loses its integrity. With the addition of a single reagent to cultures, AK phosphorylates ADP to ATP which is, in turn, used to drive the formation of light by luciferase. The AK assay has been adapted for use in high-throughput screening with Candida albicans (10–12), Cryptococcus neoformans (13, 14), and several other bacterial species (15, 16), and has successfully identified novel compounds with antifungal activity (13).

The AK assay offers advantages over traditional growth assays. First, this assay is extremely sensitive. High sensitivity is particularly attractive when considering screening compounds with intermediate activity or as complex mixtures of natural product extracts where active compounds are present in small quantities. Second, the AK assay specifically detects the lysis of fungal cells. The two main modes of action for antifungal molecules involve membrane and cell wall disruption, which are the two primary causes of cell lysis. Compounds which target or impact the fungal cell wall are particularly valuable because this essential structure is specific to fungi and lacking in the host. As such, the cell wall is an excellent target for antifungal drugs (17). Combined with the ease of use, these features make the AK assay very attractive for antifungal drug screening.

Here, we report a novel AK-release assay optimized for use with A. fumigatus. We found that this single platform allows the concurrent screening for molecules that (i) cause lysis of hyphae or (ii) prevent conidial germination. Our protocol provides a robust assay amenable to the high-throughput setting, as validated by the pilot screens described below. From these screens, we identified a cell wall active compound, PIK-75, that has activity not only against A. fumigatus, but against yeast as well, indicating that this assay is not only capable of identifying mold-active molecules but also molecules with broad-spectrum activity. Furthermore, we performed a combination screen with a subinhibitory concentration of voriconazole against a voriconazole-resistant strain of A. fumigatus and identified a compound that enhances the activity of voriconazole. Together, our data support the use of the AK assay in high-throughput screens with A. fumigatus for identification of novel, mold-active, broad-spectrum antifungal scaffolds.

## RESULTS

### The adenylate kinase assay detects lysis of A. fumigatus and is suitable for high-throughput screening applications.

As discussed above, a critical difficulty associated with screening filamentous molds for new antifungal small molecules is that changes in biomass are difficult to accurately assess and correlate with antifungal activity. A solution to this problem would be to develop an assay of antifungal activity that was independent of changes in biomass. Previously, we had used the release of intracellular AK enzyme as a reporter of antifungal and antibiotic activity using yeasts and bacteria. Therefore, we hypothesized that it could be used for high-throughput screening of a filamentous fungi such as A. fumigatus.

**FIG 2:**
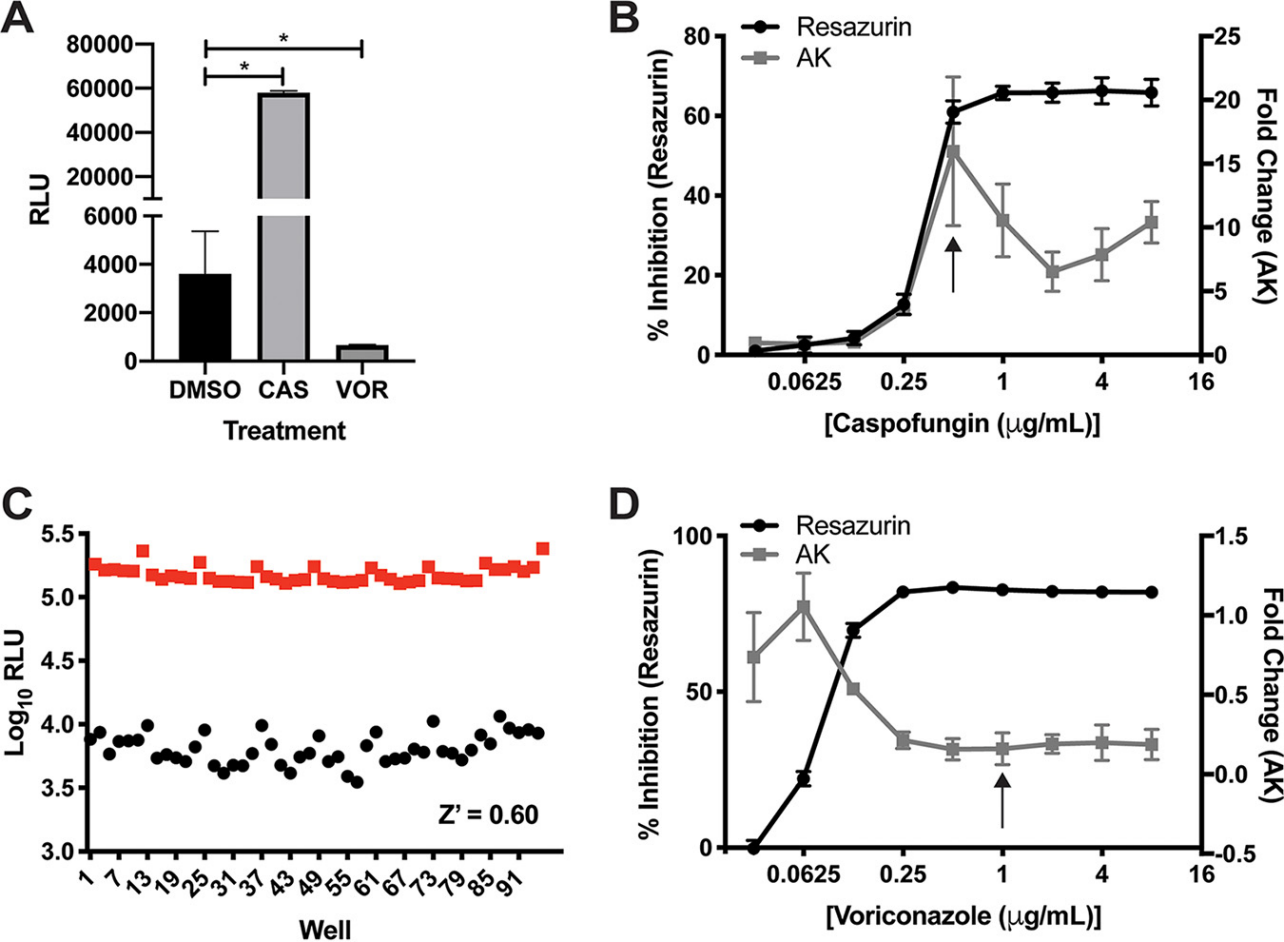
The AK assay can detect lysis and inhibition of germination of A. fumigatus. (A) Treatment of A. fumigatus strain CEA10 with 8 μg/ml caspofungin (CAS) induces lysis of A. fumigatus. Treatment with 1 μg/ml voriconazole (VOR) significantly reduces AK signal detection compared to DMSO control; *, *P* < 0.04 by one-way ANOVA with Dunnett’s multiple-comparison test. Means and standard deviations (SD) of technical triplicates are representative of two independent experiments. (B and D) AK signal compared to growth inhibition measured by resazurin over a 2-fold dilution series of caspofungin (B) or voriconazole (D). Means and SDs of technical triplicates are representative of three independent experiments. The MIC of each compound is indicated by the black arrow. (C) Representative Z’ assay of CEA10 treated with 0.5 μg/ml caspofungin in alternating columns with DMSO. Each point represents one well of the plate. Data are log transformed and are representative of at least three independent experiments completed on different days. RLU, relative luminescence units.

We have shown in yeast such as Candida albicans that fungicidal antifungal molecules cause the release of AK, while fungistatic drugs do not (12). Echinocandins induce the release of AK by inhibiting the synthesis of 1,3-β-glucan, leading to damage to the cell wall and subsequent loss of cellular integrity, resulting in lysis at the tips of A. fumigatus hyphae (18). Voriconazole, although fungicidal, does not lead to direct hyphal lysis. We therefore compared the AK activity in the medium of A. fumigatus strain CEA10 treated with voriconazole and caspofungin at concentrations above the MIC. We cultured 1 × 10^5^ conidia/ml in minimal medium containing 1% glucose and 20 mM glutamate for 16 h at 37°C and then measured AK activity in culture supernatants. Consistent with our expectations, caspofungin induced a statistically significant increase in AK signal, while voriconazole treatment did not induce lysis (Fig. 2A). Interestingly, voriconazole treatment reduced the amount of AK relative to dimethyl sulfoxide (DMSO)-treated controls. These data suggest that A. fumigatus secretes or otherwise releases AK into the growth medium during vegetative growth and that this AK release is reduced when conidia fail to germinate. Since germination is also a readout that is useful for identifying antifungal molecules active against molds, this observation indicates that the AK assay can be used to identify molecules with two distinct modes of action, i.e., those that cause fungal cell lysis and those that inhibit germination.

We next generated dose response curves using the AK assay with either caspofungin or voriconazole.

Treatment of cells with increasing concentrations of caspofungin resulted in increasing AK signal that peaks at the MIC (0.5 μg/ml) and then decreases at concentrations above the MIC (Fig. 2B). The decrease in signal above the MIC is similar to what has been observed with yeast. This phenomenon occurs because more potent drug activity reduces the number of replicating cells more quickly, resulting in fewer cells to undergo lysis and AK release over the course of growth. For this reason, the magnitude of AK signal does not correlate with the MIC of a compound. This may also be due to the paradoxical effect, where increased caspofungin concentrations trigger cell wall salvage pathways, resulting in paradoxically low antifungal activity at drug concentrations above the MIC (19, 20). Compared to the traditional growth assay with resazurin, the AK assay can detect a 3-fold increase in signal at 0.25 μg/ml caspofungin, whereas the resazurin assay only registers a 15% reduction in growth (Fig. 2B). This indicates that the AK assay detects antifungal activity of caspofungin at concentrations 2-fold lower than traditional growth assays. Cultures treated with increasing concentrations of voriconazole did not induce increased AK compared to DMSO controls; instead, the AK signal decreased in a dose-dependent manner with no further decrease in signal at 2-fold above the MIC (1 μg/ml). The sensitivity to detect inhibition of germination by voriconazole is the same between the AK and resazurin assays (Fig. 2D).

Finally, in order for an assay to be suitable for high-throughput applications, it needs to be robust enough to confidently separate real hits from noise. The statistical measure of the separation between the variability within positive and negative controls is called a Z’ score. Typically, Z’ ≥ 0.4 is considered a good assay for high-throughput screening. We measured Z’ scores of both cell lysis and inhibition of germination. For cell lysis, we used 0.5 μg/ml caspofungin and generated Z’ scores that were usually between 0.5 and 0.6 (Fig. 2C), but day-to-day variability ranged from 0.3 to 0.65. These Z’ scores fall within the range of an excellent assay for high-throughput screening (21). To measure the Z’ scores of the inhibition of germination, we used 4 μg/ml voriconazole. The Z’ scores for voriconazole were much lower, between 0.1 and 0.2, likely as a result of the much smaller separation between the voriconazole-treated group and the controls. However, our initial interest in this assay was for the detection of lysis; therefore, we continued forward with pilot screens to test our assay with A. fumigatus.

### Pilot screens validate the AK assay as a robust assay for the identification of compounds with anti-Aspergillus activity.

**FIG 3:**
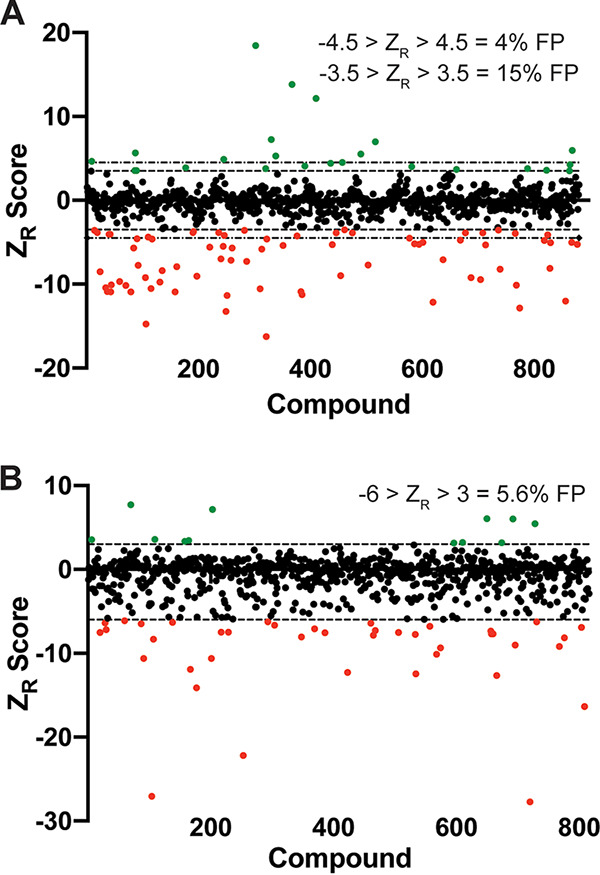
Pilot screens demonstrate good false-positive rates for the AK assay. (A and B) Screen results from the Cayman FDA library screen (A) and the NCI mechanistic set screen (B). Each data point represents the Z_R_ score of a compound in the library, with red dots indicating Z_R_ < −3.5 (A) or Z_R_ < −6 (B) and green dots indicating Z_R_ > 3.5 (A) or Z_R_ > 3 (B). False-positive (FP) rates are given for each threshold cutoff. (A) The dashed lines delineate −3.5 and 3.5. The dot and dash lines delineate −4.5 and 4.5. (B) Dashed lines delineate −6 and 3.

FDA-approved drug libraries are valuable resources in antifungal screen development because they contain known antifungals and many of these libraries have already been screened against fungal organisms. Therefore, these screens act as a proof-of-concept to determine whether an assay can identify compounds with known activity. We performed a screen with a library of 875 FDA-approved drugs at 25 μM. Screening data was log transformed and then the robust Z (Z_R_) score was calculated for each well (22). The Z_R_ score is a measure of how many absolute deviations the signal from each well is from the median of the plate. To determine appropriate thresholds for calling hits, we compared the false positive rates between two Z_R_ thresholds. First, we included all hits with −3.5 < Z_R_ < 3.5. This analysis yielded 24 compounds that induced lysis (Z_R_ > 3.5) and 74 compounds that reduced AK signal (Z_R_ < −3.5) (Fig. 3A, Table 1). In total, 22 of the 98 hits were antifungals and 39 have described antifungal activity, therefore we performed secondary validation with 35 of the remaining compounds by testing them under the exact same conditions as the screen. Of these 35, 21 compounds were validated as true hits, giving a false-positive rate of 14/35 or 40%. To determine an estimated false-positive rate for the entire screen, we included the antifungal compounds and those with previously reported antifungal activity, which gives a false-positive rate of 15% (14/96). Increasing the stringency of calling hits to −4.5 > Z_R_ > 4.5 decreases the false-positive rate to 3/19 (16%) for compounds without antifungal activity or 3/80 (4%) for all hits (Fig. 3A).

**TABLE 1:** Hits from FDA drug repurposing screen against A. fumigatus^*b*^

Drug	Z_R_ score	Antifungal activity
Floxuridine	–8.4	✓(49)
Disulfiram	–13.23	✓(25, 49)
Ceritinib	4.5	
Telmisartan	–4.05	
5-FOA	–7.92	✓(50)
Dronedarone	–5.03	✓(14)
Flecainide	–7.72	✓(51)
Sulfamethoxazole	–7.1	✓(52)
Bleomycin	–10.5	✓(53)
Dapsone	–9.2	
Trimipramine	18.45	
Sulfanilamide	–3.88	✓^*a*^
Natamycin	–4.05	✓ ^*a*^
5-Fluorocytosine	–10.91	✓^*a*^
Amphotericin B	–10.1	✓ ^*a*^
Itraconazole	–4.58	✓ ^*a*^
Posaconazole	–3.85	✓ ^*a*^
Ketoconazole	–5.47	✓ ^*a*^
Clotrimazole	–4.12	✓ ^*a*^
Miconazole	–5.68	✓ ^*a*^
Voriconazole	–3.59	✓ ^*a*^
Ciclopirox	–10.56	✓ ^*a*^
Micafungin	12.14	✓ ^*a*^
Oxiconazole	–5.29	✓ ^*a*^
Naftifine	–8.99	✓ ^*a*^
Griseofulvin	–3.52	✓ ^*a*^
Anidulafungin	6.98	✓ ^*a*^
Sertaconazole	–5.03	✓ ^*a*^
Tavaborole	–12.16	✓ ^*a*^
Butenafine	–5.33	✓ ^*a*^
Efinaconazole	–8.21	✓ ^*a*^
Isavuconazonium	–10.12	✓ ^*a*^
Terbinafine	–5.23	✓ ^*a*^
Mebendazole	–3.88	✓(51)
Albendazole	–4.74	✓(49, 51)
Nitazoxanide	–9.21	
Pimozide	7.25	✓(14)
Chlorpromazine	–4.62	✓(26)
Aripiprazole	–3.88	✓(54)
Asenapine	–8.12	
Abacavir	–3.72	
Nelfinavir	–6.99	
Amiodorone	4.88	✓(14, 24)
Ponatinib	–8.5	✓(55)
Dabrafenib	13.82	
FK-506 (Tacrolimus)	–12.01	✓
C*y*closporin A	–10.13	✓
Pimecrolimus	–10.91	✓
Cinacalcet	–5.82	
5-Azacytidine	–3.84	
Hexachlorophene	–3.95	✓(14)
A-771726	–10.92	
Methotrexate	–4.63	✓(56)
Mitoxantrone	–9.03	✓(57)
Doxorubicin	–5.57	✓(57)
Carbidopa	–3.88	
Apomorphine	–16.26	✓(58)
Trifluoperazine	–3.89	✓(14, 59)
Panobinostat	3.55	✓(60)
Mycophenolic acid	–5.12	✓(61)
Phenelzine	–12.84	✓(62)
Temsirolimus	–10.41	✓
Everolimus	–10.87	✓
Rapamycin	–7.74	✓
Droxidopa	–9.44	
Meclofenamate	–4.79	
Clomiphene	3.77	✓(14)
Verteporfin	–10.89	
Colistin	–4.25	✓(63)
Oleoyl ethanolamide	–4.11	
6-Thioguanine	–7.27	
Fingolimod	–9.7	✓(64)
Vortioxetine	3.7	
Pitavastatin	–7.15	✓
Fluvastatin	–5.02	✓
Atorvastatin	4.64	✓
Lovastatin	5.97	✓
Mitotane	4.41	
Eltrombopag	5.64	✓(65)
Auranofin	–11.33	✓(66)
Imatinib	–5.68	
Axitinib	–14.74	
Febuxostat	–9.74	
Dopamine	–5.23	✓(67)

a On-label antifungal drugs.

b Robust Z scores from the screen are listed and drugs with previously reported antifungal activity are marked with ✓.

Notably, our screen identified all antifungal compounds except fluconazole, which is not active against A. fumigatus (23). In addition to antifungal drugs, our screen also identified several compounds with previously reported activity against A. fumigatus, including albendazole (24), disulfiram (25), chlorpromazine, trifluoperazine (26), amiodarone (24), sulfamethoxazole (24), dopamine (24), doxorubicin, and mitoxantrone (24). Furthermore, several other drugs were identified that have been previously reported from at least one repurposing screen with yeasts (Table 1). Together, the results of this FDA repurposing screen verify that our screening assay can identify compounds with known antifungal activity.

**FIG 4:**
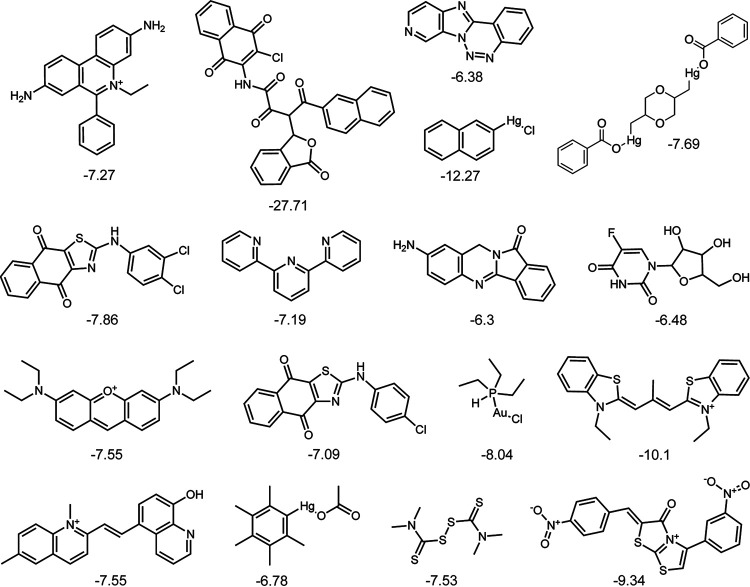
Compounds with an MIC below 20 μM against A. fumigatus. Structures of 17 compounds from the NCI mechanistic set that inhibited germination at 20 μM, as determined by microscopic analysis of each well. Shown below each compound is the Z_R_ score from the screen.

As an additional validation, we performed a pilot screen using the National Cancer Institute (NCI) mechanistic set IV, a library of 812 compounds, at a concentration of 20 μM. We calculated the robust Z (Z_R_) score of each well (22) and hits were called using threshold scores of −6 > Z_R_ > 3. With these criteria, we identified 41 compounds with Z_R_ < −6 and 12 compounds with Z_R_ > 3 (Fig. 3B). We performed secondary screening and 51 of 53 compounds showed the same pattern of AK signal as the original screen, for a 5.6% false-positive rate. Additionally, we evaluated each well with Z_R_ < −6 microscopically to determine how often this decrease in AK signal is indicative of antifungal activity. Of 41 tested compounds, 17 inhibited germination (Fig. 4), 6 had an intermediate growth phenotype, and the remaining 18 compounds had no apparent effect on growth. The decrease in AK signal from the remaining compounds that had no effect on growth could be a result of inhibition of luciferase, the adenylate kinase in the assay, or of the secretion/release of AK from the fungus itself. There are several known compound families that are inhibitors of luciferase (27), therefore, in subsequent screens, we included an additional secondary screening assay where wells were inoculated with A. fumigatus and incubated without compound, after which the compound was added just before the addition of the AK detection reagent (AKDR). The compounds identified in this screen included some with known anti-Aspergillus activity, including tamoxifen (unpublished results, Damian Krysan) and the disulfiram analog thiram (25, 28). Furthermore, 17 compounds inhibited germination, indicating that 33% of hits have an MIC of <20 μM (Fig. 4). The chemical structures of these compounds were not of interest to us for development as antifungals; therefore, we did not follow up on any of them. These pilot screens give us confidence that the AK assay could reliably identify compounds with anti-Aspergillus activity that induce hyphal lysis or inhibit conidial germination.

### Screening a small protein kinase inhibitor library identifies a molecule that disrupts cell wall integrity in A. fumigatus.

**FIG 5:**
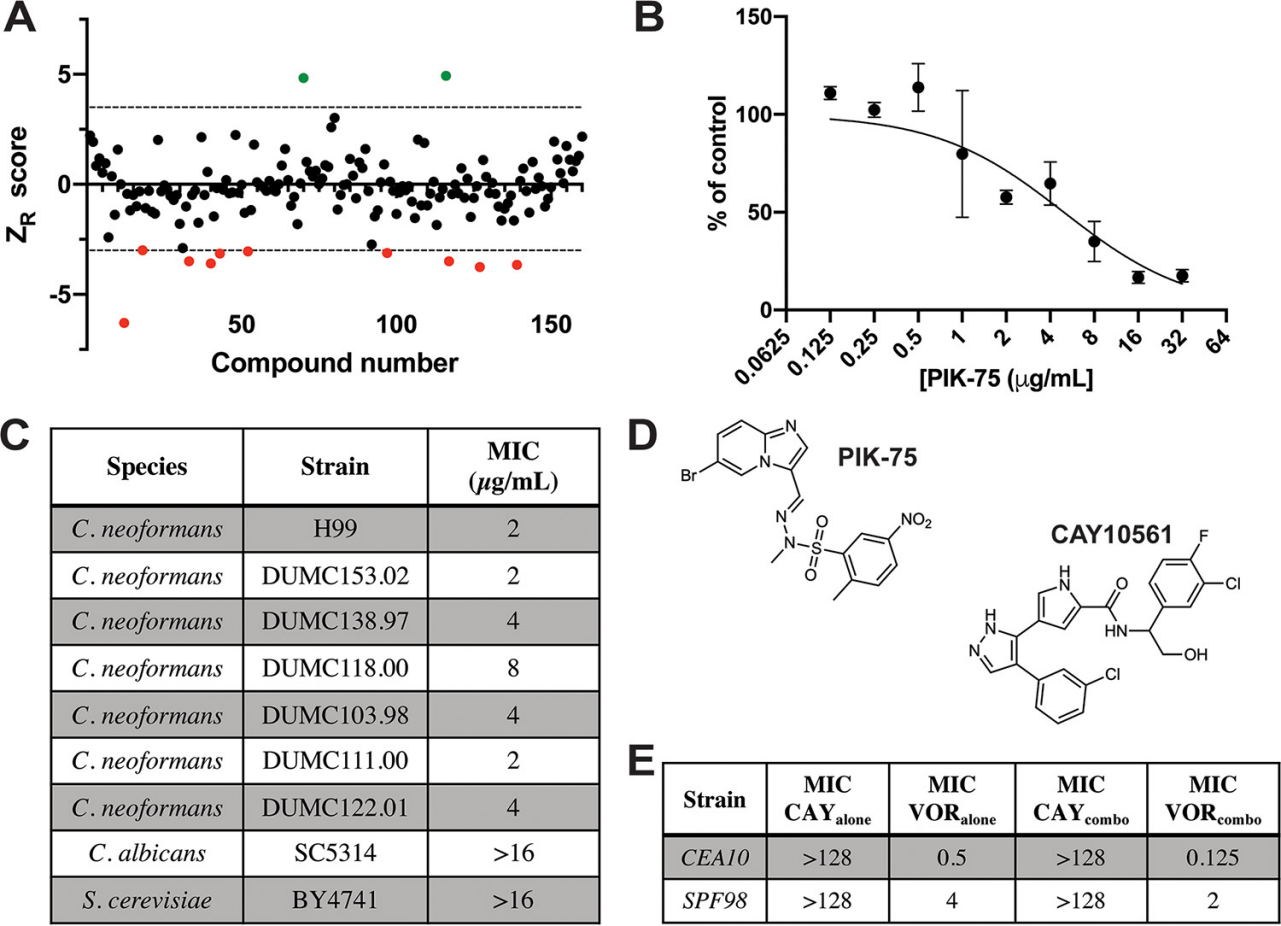
A screen of kinase inhibitors identifies novel molecules with antifungal activity. (A) Robust Z scores (Z_R_) of kinase inhibitor library screen against A. fumigatus CEA10. Dashed lines represent Z_R_= ±3.5. Red dots are compounds with Z_R_ < −3.5, and green dots are Z_R_ > 3.5. (B) A. fumigatus CEA10 was grown for 14 h and then treated with a 2-fold dilution series of PIK-75 for 24 h. Data are presented as % of control, based on resazurin. Means and SDs of four technical replicates are representative of three independent experiments. (C) MICs of PIK-75 against yeast strains. (D) Structures of PIK-75 and CAY10561. (E) Fractional inhibitory concentration (FIC) results between CAY10561 (CAY) and voriconazole (VOR). Data are representative of two independent experiments.

Protein kinases regulate about ∼30% of biological processes in cells (29), including essential processes such as growth, cell cycle, and stress response, all of which underlie pathogenesis. Therefore, kinases make attractive antifungal drug targets. To test the ability of our assay to detect novel antifungal compounds, we performed a screen with a library of 160 kinase inhibitors at 25 μM. We identified 12 candidate molecules using −3 > Z_R_ > 3 as a threshold to call hits (Fig. 5A). Of these molecules, 10 were validated as true hits (2/12 = 17% false positive) and none inhibited AK activity by adding the drug to cultures at the time of AKDR addition. We tested the MIC of each compound and found that only one inhibited germination at the tested concentrations. This compound, chelerythrine, has known broad-spectrum antifungal activity (30, 31). While none of the remaining compounds completely inhibited germination at tested concentrations, one compound, the PI3K inhibitor PIK-75 (Z_R_ = 4.9, Fig. 5D), reduced growth visibly at 12 μM. PIK-75 also inhibited growth of A. fumigatus when added to pregrown hyphal cultures, as measured by resazurin (Fig. 5B). Importantly, we tested the activity of PIK-75 against the yeasts Candida albicans, Cryptococcus neoformans, and Saccharomyces cerevisiae and found that PIK-75 has activity against a C. neoformans laboratory reference strain and clinical isolates, with MICs between 2 and 8 μg/ml (Fig. 5C). This result suggests that our assay is capable of identifying broad-spectrum antifungals, not only those that are active against molds.

**FIG 6:**
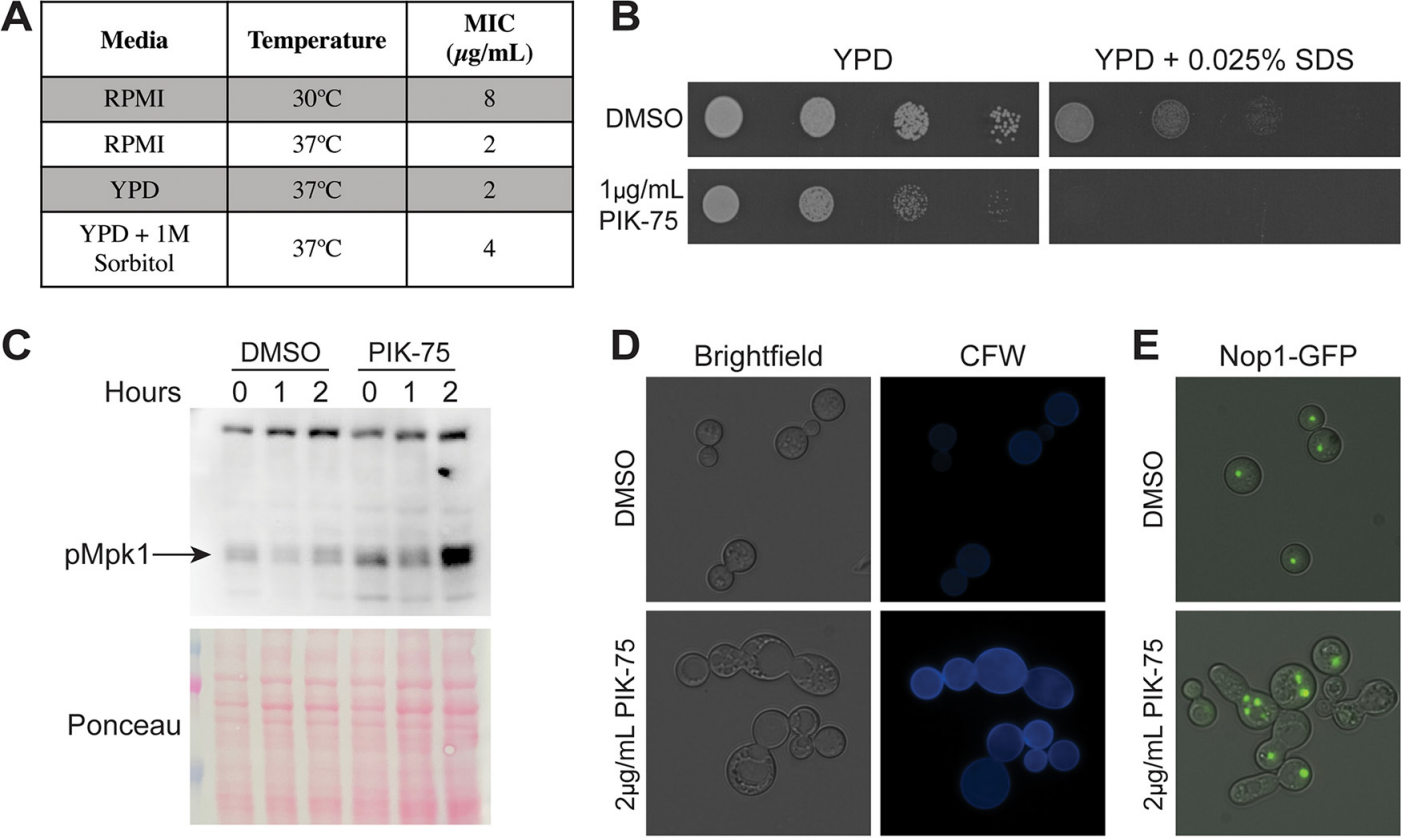
PIK-75 is a cell wall active compound that induces cell cycle defects. (A) MICs of PIK-75 against C. neoformans H99 at 30°C versus 37°C and YPD ± 1 M sorbitol. (B) Spot dilutions of H99 on YPD containing 1 μg/ml PIK-75 with 0.025% SDS. Plates were incubated at 37°C for 48 h. (C) Phosphorylated Mpk1 Western blot of cells treated with DMSO or 4 μg/ml PIK-75, and then challenged with CFW for 0, 1, or 2 h. Ponceau staining is shown as loading control. (D) Brightfield and CFW staining of H99 treated with 2 μg/ml PIK-75 for 24 h at 37C. (E) Merged image of brightfield and GFP channels of H99^Nop1-GFP^ cultured under the same conditions as in panel C. All images were taken using the same exposure and are representative of at least three independent experiments.

PIK-75 was one of the two compounds that induced lysis of A. fumigatus. Given that cell lysis is often mediated by loss of cell wall integrity, we tested the hypothesis that the lytic activity of PIK-75 is cell wall mediated. To test this, we used C. neoformans, due to the more potent activity against this organism. First, we tested the MIC of PIK-75 against C. neoformans in the presence and absence of 1 M sorbitol, an osmostabilizer that helps protect cells from death upon loss of cell integrity. As expected for a cell wall active compound, the MIC of PIK-75 increased 2-fold in the presence of sorbitol (Fig. 6A). Furthermore, the activity of PIK-75 is dependent on temperature, with the MIC increasing 4-fold at 30°C compared to 37°C (Fig. 6A). Next, we tested the interaction between PIK-75 and cell wall stressors. We observed a strong synergy with SDS, where growth is almost completely inhibited at a concentration of SDS and PIK-75 that have only moderate effects on growth individually (Fig. 6B). This interaction, as well as our MIC data, suggests that the antifungal activity of PIK-75 is due, at least in part, to loss of cell wall integrity.

PIK-75 is known as a class I PI3K inhibitor; however, fungi lack class I PI3Ks (32), so the activity of PIK-75 must be mediated through inhibition of another kinase(s). A screen of the mammalian kinome identified 27 kinases that are strongly inhibited by PIK-75, including protein kinase C (PKC) (31), a serine-threonine kinase. PKC has a well-established role in the fungal cell wall integrity pathway (33), thus we hypothesized that the cell wall integrity defects could be due to inhibition of PKC. To test this, we treated C. neoformans with the cell wall stressor calcofluor white (CFW) in the presence and absence of PIK-75 and monitored Mpk1 phosphorylation by Western blotting. Contrary to what we expected, we observed an increase in Mpk1 phosphorylation after 2 h of CFW exposure in PIK-75-treated cells compared to controls (Fig. 6C). Consistent with increased Mpk1 phosphorylation, we observed an increase in chitin content on the surface of cells treated with PIK-75, as visualized by calcofluor white staining (Fig. 6D). Upon microscopic analysis, we also observed morphological defects in cells treated with PIK-75. Treated cells displayed elongated and enlarged morphology and often were present as chains of cells that failed to separate. Septa were sometimes, but not always, present between these cells (Fig. 6D). These morphological phenotypes are classically associated with cell cycle defects (34); therefore, we tested whether these morphologically abnormal cells also harbored abnormal nuclear phenotypes. Using a strain bearing a green fluorescent protein (GFP)-tagged nucleolar protein, Nop1, we observed that, compared to control cells which each contained one Nop1 punctum, these elongated chains of cells often contained cell bodies with multiple Nop1 puncta and other cell bodies with a complete absence of Nop1 (Fig. 6E). Together, these data suggest cell cycle defects induced by PIK-75 treatment.

### Screening a voriconazole-resistant isolate with a protein kinase library identifies a modulator of voriconazole resistance.

The emergence of antifungal resistance of A. fumigatus to azole drugs is becoming increasingly common worldwide (2, 35). Identifying compounds that are active against azole-resistant strains or that enhance the activity of azoles against these strains is key in combatting this growing clinical problem. To test the efficacy of our screen to identify compounds that modulate voriconazole activity, we performed a combination screen with the kinase inhibitor library at 25 μM and a subinhibitory concentration of voriconazole (1 μg/ml) against a voriconazole-resistant clinical isolate of A. fumigatus, SPF98. We identified 6 compounds with Z_R_ > 3 and 13 compounds with Z_R_ < −3 for a total of 19 hits. Of these 19 hits, 14 were validated as real hits (25% false- positive rate) and one compound inhibited the AK signal when added simultaneously with the AKDR. In addition to 8 compounds that were identified in our reference strain screen, we also identified 6 unique hits through the combination screen. We tested the MICs of each compound in combination with voriconazole and found one compound, CAY10561 (Fig. 5D), which inhibited germination completely at 100 μM (46 μg/ml) in the presence of voriconazole. This compound did not have any antifungal activity at tested concentrations (>128μg/ml); however, using checkerboard assays to assess drug interactions, we observed a 2-fold decrease in voriconazole MIC at 8 μg/ml CAY10561 against SPF98 and a 4-fold decrease in voriconazole MIC at 32 μg/ml CAY10561 against voriconazole-sensitive CEA10 (Fig. 5E). CAY10561 is an ERK2 (MAPK) inhibitor (36), however, we did not observe any effect of drug treatment on induction of Mpk1/Rlm1 cell wall integrity pathway (CWIP) genes in response to Congo red treatment (Fig. S1 in the supplemental material). Our combination screen identified a compound that enhances the activity of voriconazole against a resistant strain, supporting the use of this assay to identify novel compounds with synergistic interactions with voriconazole to combat emerging azole resistance in A. fumigatus clinical isolates.

10.1128/mSphere.00539-21.1 Figure S1 Mpk1/Rlm1 target gene expression is not affected by CAY10561 treatment. A. fumigatus CEA10^Δ^*^ku80^* was cultured for 14 h and then treated with DMSO or 32 μg/ml CAY10561. Cultures were treated with 500 ng/ml Congo red for 1 h. Gene expression is normalized to samples taken before addition of Congo red. Means and SDs are representative of three biological triplicates. Download FIG S1, TIF file, 2.3 MB. Copyright © 2021 Beattie and Krysan. 2021 Beattie and Krysan. https://creativecommons.org/licenses/by/4.0/ This content is distributed under the terms of the Creative Commons Attribution 4.0 International license.

## DISCUSSION

There is an urgent need for antifungal drugs with potent mold activity to fight the fatal infections caused by these organisms. Here, we adapted the AK assay as an alternative screening platform to traditional growth assays for high-throughput screening with A. fumigatus. The work reported here demonstrates that this assay is an innovative way to detect novel anti-mold scaffolds with a variety of mechanisms of action.

The dual readout of this assay is a unique aspect of the AK assay when used with A. fumigatus. Adenylate kinase and several other metabolic enzymes have been detected in the culture supernatant of Aspergillus brasiliensis (37), indicating release of these enzymes during vegetative growth, which likely accounts for the much higher baseline AK signal in A. fumigatus cultures compared to yeast (11). The ability to harness this increased signal as an additional method to identify mold-active compounds gives more flexibility to the assay and increases the number of potential antifungals that can be identified in a single screen. Despite the low Z’ scores for germination inhibition and the other possible routes for decrease in AK signal, our pilot screens successfully identified compounds which inhibited germination. Of the compounds that reduced the AK signal in our screen of the NCI mechanistic set, 33% inhibited germination, yielding 17 compounds with an MIC below 20 μM. Thus, this screening platform offers an additional advantage when used with A. fumigatus in that it allows identification of compounds with mechanisms of actions besides indirect or direct cell lysis.

To validate this assay in a screening setting, we performed several pilot screens, including use of an FDA library containing many known antifungal drugs. Overall, these screens had good false-positive rates, ranging from 5 to 20%, and, importantly, known antifungals were identified as validated hits. As demonstrated in Fig. 3, the false-positive rates of a screen will change based on where you set the threshold to call hits. Several factors may influence the stringency of your threshold, one of the most important being the size of the library being screened. Another important part of validating this screening platform is the ability to identify known antifungals. Using a library of FDA-approved drugs, we identified 23 of 24 antifungal drugs. Importantly, our screen failed to identify fluconazole, which does not have activity against A. fumigatus. We also identified several compounds with previously reported antifungal activity, several of which have recently been identified in repurposing screens. These results support the specificity of the assay to detect compounds with antifungal activity and not just drugs and drug-like compounds. It is important to keep in mind that the AK signal generated in this assay does not correlate with the MIC of a compound; thus, this assay is purely for compound discovery, not compound characterization. Compounds with low activity can generate high AK signal and vice versa. We view this as an advantage of the assay because it allows detection of even very weakly active molecules. However, it is important to include the appropriate secondary screening and characterization of hits to evaluate antifungal activity.

With a small library of kinase inhibitors, we identified PIK-75 which lyses A. fumigatus hyphae and importantly also has activity against yeast. Our goal is to develop mold-active compounds, however, the extensive molecular tools available in yeasts greatly improve the ability to identify the mechanism(s) of action, and broad-spectrum compounds are more attractive for clinical use. Although PIK-75 is not an ideal candidate for development into a clinical antifungal, it is a great proof-of-concept compound for our pilot screen. Cell lysis often occurs as a result of loss of cell wall integrity; therefore, the AK assay is optimal for identifying cell wall-active compounds (13). The temperature dependence and the ability of sorbitol to suppress the activity of PIK-75 indicate that the activity of this compound is cell wall mediated. Indeed, we observed that Mpk1 is hyperphosphorylated with PIK-75 treatment. Hyperactivation of the CWIP can paradoxically mimic the effects of inhibition of the pathway (38), which is consistent with the observed phenotypes indicating loss of cell wall integrity. Increased activation of the cell wall pathway in PIK-75-treated cells is indicative of an indirect effect on the cell wall rather than direct inhibition.

The cell cycle and the cell wall pathway are two processes that must be carefully coordinated in order for proper cellular development. The site of bud formation requires extensive cell wall remodeling and synthesis for the emergence and generation of the daughter cell, and chitin synthesis is required for primary septum formation between mother and daughter cells. Thus, the cell wall synthesis genes are expressed differentially throughout the cell cycle (39, 40) and the cell wall integrity pathway is activated at a distinct point during the cell cycle (41, 42). The mammalian kinome studies of PIK-75 identified CDK7, CDK9, and CDK14 as targets of PIK-75 (43). Our data support a role of PIK-75 in perturbation of the cell cycle, as observed with the morphological and nuclear abnormalities suggesting a cell cycle kinase as a potential target of this drug. It is also possible that PIK-75 is targeting multiple kinases in the cell, as kinase inhibitors tend to be promiscuous and bind multiple kinases.

Our results with PIK-75 are an excellent example of how the AK assay can identify cell wall active compounds but is not limited to those that act on the cell wall directly. Together with the ability to identify compounds that inhibit germination, this assay can yield a variety of hits with different mechanisms of action, but in a more targeted and sensitive way than standard growth assays. The potential for the AK assay extends beyond just A. fumigatus. Other organisms, such as Fusarium spp., *Mucor* spp., *Rhizopus* spp., and *Scedosporium* spp., while less common than A. fumigatus, are becoming more prevalent in susceptible patient populations (44). These organisms are intrinsically resistant to many, and sometimes all, antifungals (45). For this reason, the mortality rates of these infections remain exceptionally high, even with treatment (44). Application of the AK assay is theoretically possible for any species and we have preliminary results that suggest this platform is applicable to other molds. This work has laid a solid foundation for the development of novel anti-mold scaffolds. Currently we are applying this platform to much larger and more diverse chemical libraries and have already had success in identifying compounds with promising activities. We strongly believe that the diversification of screening platforms and organisms will yield novel antifungal compounds for the treatment of these fatal mold infections.

## MATERIALS AND METHODS

### Strains, media, and chemicals.

All yeast strains were maintained on yeast extract-peptone-dextrose (YPD) medium from 25% glycerol stocks stored at −80°C. All A. fumigatus strains were maintained on glucose minimal media (GMM) (44) from 25% glycerol stocks stored at −80°C. A. fumigatus strain CEA10 was a gift from Robert Cramer (Dartmouth College), strains SPF98 and CEA10^Δ^*^ku80^* were a gift from W. Scott Moye-Rowley (University of Iowa), the C. neoformans DUMC clinical isolate series was a gift from John Perfect (Duke University). Glucose screening medium (GSM) is a modified GMM prepared as a 2× minimal medium base (1.04 g/liter KCl, 1.04 g/liter MgSO_4_·7H_2_O, 3.04 g/liter KH_2_PO_4_ monobasic, 4.4 mg/liter ZnSO_4_·7H_2_0, 2.2 mg/liter H_3_BO_3_, 1 mg/liter MnCl_2_·4H_2_O, 1 mg/liter FeSO_4_·7H_2_O, 0.32 mg/liter CoCl_2_·5H_2_O, 0.32 mg/liter CuSO_4_·5H_2_O, 0.22 mg/liter (NH_4_)6Mo_7_O_24_·4H_2_O, 10 mg/liter Na_4_EDTA, 5.88 g/liter l-glutamic acid [pH 6.5]) combined with 20% glucose solution for final concentration of 1% glucose and 1× minimal medium base.

NCI mechanistic set IV (plates 4847 to 4857) was obtained from the National Institutes of Health National Cancer Institute Developmental Therapeutics program (NIH NCI DTP). The Kinase Screening Library (item no. 10505) and FDA-Approved Drug Screening Library (item no. 23538) were obtained from Cayman Chemicals. PIK-75 (S1205) was obtained from Selleck Chemicals. CAY10561 (10010043) was obtained from Cayman Chemicals.

### AK optimization.

To compare AK and resazurin, plates were inoculated with 1 × 10^5^ conidia/ml in GSM, with a 2-fold dilution series of caspofungin (Sigma, SML0425) or voriconazole (Sigma, PZ0005) with 0.32% dimethyl sulfoxide (DMSO) in all wells. Plates were incubated at 37°C for 18 h, then brought to room temperature for 1.5 h. AK detection reagent (100 μl) (Toxilight non-destructive cytotoxicity bioassay, Lonza, LT07) was added to each well, then plates were incubated for 1 h at room temperature and luminescence was measured with 140 ms integration on a SpectraMax i3X Multi-Mode plate reader (Molecular Devices). For resazurin assays, 0.001% resazurin was added to each well at inoculation, then, after incubation, fluorescence was measured with excitation at 570 nm and emission at 615 nm. Each assay was performed in technical triplicate and replicated on three independent days. Z’ scores were measured by filling plates with GSM with either 0.5 μg/ml caspofungin or 4 μg/ml voriconazole, alternating columns with DMSO controls. All wells were inoculated with 1 × 10^5^ CEA10 conidia/ml and plates were incubated for 16 h at 37°C and AK assay was developed as described above. Data were log transformed and Z’ scores were calculated as previously described (21). All Z’ scores were measured on at least 3 independent days.

### Small molecule screens.

The NCI small molecule screen was performed by dispensing 80 μl of screening medium into plates containing compound, then dispensing 20 μl of conidial suspension (0.01% Tween 80 with 5 × 10^5^ conidia/ml) for final concentrations of 20 μM compound, 2% DMSO, and 1 × 10^5^ conidia/ml. Cayman kinase inhibitor and FDA-approved libraries were screened at 25 μM compound, 1% DMSO and 1 × 10^5^ conidia/ml concentrations. Plates were incubated at 37°C for 16 h, then allowed to come to room temperature for 1.5 h before addition of 100 μl AK detection reagent per well (Toxilight non-destructive cytotoxicity bioassay, Lonza, LT07). Plates were incubated for 1 h at room temperature, then luminescence was measured with 140 ms integration on a SpectraMax i3X Multi-Mode plate reader (Molecular Devices). Data were log transformed and Z_R_ scores for each well were calculated as previously described (22). Hits were validated by testing compounds under the same screening conditions, with DMSO controls filling the remainder of the plates. For Cayman libraries, hits were also validated by growing wells without compound using the above conditions, then adding compound just before addition of AK detection reagent. Data were log transformed and Z_R_ scores were calculated for each well (22).

### MIC and FIC checkerboard assays.

MICs were determined using CLSI guidelines (46, 47). All yeasts were cultured overnight in 3 ml YPD at 30°C, then washed twice in sterile phosphate-buffered saline (PBS). Two-fold serial dilutions of each drug were prepared in RMPI + MOPS pH 7 (Gibco RPMI 1640 with l-glutamine [11875-093] and 0.165 M MOPS), then 1 × 10^3^ cells were added per well. Plates were incubated at 37°C for 24 (C. albicans and S. cerevisiae) or 72 (C. neoformans) hours. For A. fumigatus, wells were inoculated with 1.25 × 10^3^ conidia in RPMI + MOPS pH 7. Plates were incubated at 37°C for 48 h. MICs with sorbitol were performed in YPD or YPD + 1 M sorbitol. For fractional inhibitory concentrations, we performed standard checkerboard dilution assays (48). Briefly, one drug was diluted in a 2-fold series across a 96-well plate and the other drug was diluted in a 2-fold series down a 96-well plate, then conidia (1.25 × 10^3^) or yeast (1 × 10^3^) were added to each well for a total volume of 200 μl RPMI + MOPS pH 7. The MIC of each drug alone and each drug in combination was determined. Fractional inhibitory concentration index was determined by the equation: (MIC_A alone_/MIC_A combo_) + (MIC_B alone_/MIC_B combo_). Each assay was done in technical duplicate and at least two independent experiments were performed on different days.

### Growth assays.

PIK-75 activity against A. fumigatus hyphae was measured by inoculating a 96-well plate with 100 μl of RPMI + MOPS pH 7 with 1 × 10^3^ CEA10 conidia. Plates were incubated at 37°C for 14 h, then 100 μl of RPMI + MOPS pH 7 with a 2-fold dilution series of PIK-75 was added to each well for a highest final concentration of 32 μg/ml. Resazurin was added to each well for a final concentration of 0.002%, then plates were incubated for an additional 24 h. After incubation, fluorescence was measured with excitation of 570 nm and emission of 615 nm. The assay was performed with four technical replicates on at least three independent days. PIK-75 interaction with SDS was performed by diluting an overnight culture of C. neoformans H99 to an optical density at 600 nm (OD_600_) of 0.1, then making three 10-fold dilutions and spotting 5 μl of each dilution on YPD, YPD + 1μg/ml PIK, YPD + 0.025% SDS, or YPD + 1μg/ml PIK-75 + 0.025% SDS. Plates were incubated at 37°C for 48 h and imaged with an Epson scanner.

### Microscopy.

For microscopy experiments, overnight cultures of C. neoformans strains H99 or H99^Nop1-GFP^ were diluted to 1 × 10^6^ CFU/ml in 3 ml YPD with 2 μg/ml PIK-75 or DMSO (0.02%) and incubated for 24 h at 37°C. Cells were washed three times in sterile PBS, stained with 20 μg/ml CFW in PBS (Fluorescent brightener 28, Sigma, F3543), and incubated for 20 min at RT in the dark. Cells were imaged on a Nikon epifluorescence microscope with a Cool Snap HQ2 camera and Nikon Elements image acquisition and analysis software. Images were processed in Photoshop only to increase ease of viewing. All images were adjusted equally.

### Gene expression.

Inoculums of 1 × 10^7^ CEA10*^Δku80^* conidia/ml were inoculated in GMM and incubated for 14 h at 37°C with shaking. Cultures were treated with 32 μg/ml CAY10561 or 0.32% DMSO and incubation at 37°C with shaking was continued for 2 h. Samples were taken from each culture, then 500 ng/ml Congo red (Sigma, C6767) was added to each culture and cultures were incubated for 1 h at 37°C with shaking. Mycelia were collected with vacuum filtration and immediately frozen with liquid nitrogen. Tissue was lyophilized and then ground with glass beads with a bead-beater. RNA was extracted in 1 ml of TRIazole solution (Life Technologies, 15596026), then 200 μl of chloroform was added, and the aqueous phase was collected after centrifugation. The aqueous phase was mixed with an equal volume of isopropanol and pelleted by centrifugation. RNA (5 μg) was treated with TURBO DNA-free kit (Invitrogen, AM1907) according to the manufacturer’s protocol. DNase-treated RNA (500 ng) was used for cDNA synthesis with iScript cDNA synthesis kit (Bio-Rad, 1708891), then cDNA was diluted 1:5 with ddH_2_O. Real-time quantitative PCR (RT-qPCR) analysis was performed in 20-μl reactions using 2 μl of dilute cDNA per reaction with iQ SYBR green Supermix (Bio-Rad). Samples were run on a CFX96 Real-Time system (Bio-Rad) and genes were normalized to *tubA* expression. Gene expression was performed in biological triplicate. Primers used in this study are listed in Table S1.

10.1128/mSphere.00539-21.2 TABLE S1 Primers used in this study. Download Table S1, DOCX file, 0.01 MB. Copyright © 2021 Beattie and Krysan. 2021 Beattie and Krysan. https://creativecommons.org/licenses/by/4.0/ This content is distributed under the terms of the Creative Commons Attribution 4.0 International license.

### Western blotting.

An overnight culture of H99 was diluted into 100 ml YPD and cultures were incubated at 30°C for 1.5 h with shaking at 200 rpm, after which 4 μg/ml or 2 μg/ml of PIK-75 or DMSO (0.04%) was added to cultures and incubation was continued for 1.5 h at 30°C. A sample of cells at OD_600_ ∼10 was collected for the *t* = 0 time point, then 200 μg/ml CFW (Fluorescent brightener 28, Sigma, F3543) was added to each culture and cultures were shifted to 37°C with shaking at 200 rpm. Samples of cells at OD_600_ ∼10 were taken at 1 and 2 h after CFW addition. Cell pellets were snap-frozen and stored at −80°C. Protein was extracted in extraction buffer (10 mM HEPES pH 7.4 to 7.9, 1.5 mM MgCl_2_, 10 mM KCl, 1 mM dithiothreitol [DTT], 1× HALT protease and phosphatase inhibitor cocktail) by five bead beating cycles of 30 s followed by 30 s on ice per cycle. Supernatant was removed from the glass beads and the protein concentration was quantified by Bradford assay. Protein (15 μg) was loaded on a 10% SDS-PAGE gel and run at 80V. Samples were transferred to nitrocellulose membrane for 1 h at 100V, then membrane was stained with Ponceau for 15 min at RT. The membrane was blocked with 5% bovine serum albumin (BSA) in Tris-buffered saline with Tween 20 (TBST)for 1 h at RT, then the membrane was incubated with 1:2,000 rabbit anti-p-p44 (Cell Signaling, Phospho-p44/42 MAPK, number 4370) in 5% BSA/TBST. The membrane was washed 3 times with TBST, then incubated for 1 h at RT with 1:20,000 goat anti-rabbit horseradish peroxidase (HRP) (Bio-Rad, STAR208P). The blot was developed with chemiluminescent substrate.
