# Supplementary figures and images for: A Unique Dual-Readout High-Throughput Screening Assay To Identify Antifungal Compounds with Aspergillus fumigatus
Source: mSphere. 2021 Aug 18;6(4):e00539-21. doi: 10.1128/mSphere.00539-21 (PMC8386399; doi:10.1128/mSphere.00539-21)

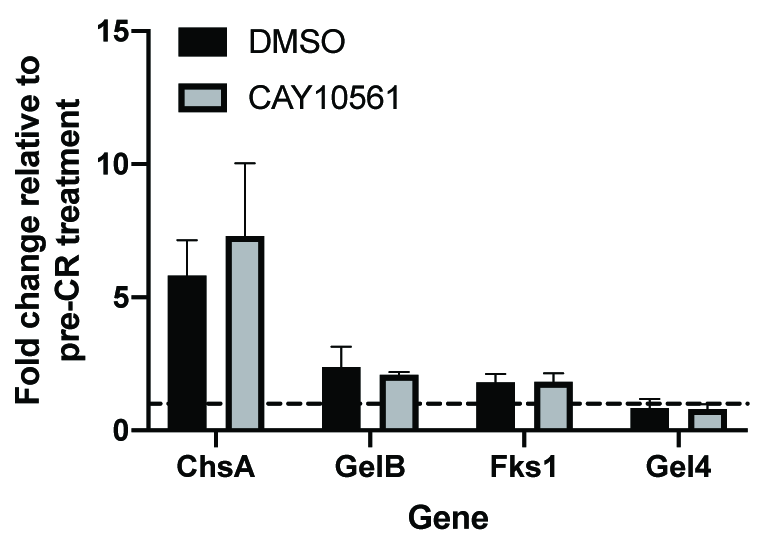

Supplement: Figure S1 [file msphere.00539-21-sf001.tif]
