# Supplementary material for: A Unique Dual-Readout High-Throughput Screening Assay To Identify Antifungal Compounds with Aspergillus fumigatus
Source: mSphere. 2021 Aug 18;6(4):e00539-21. doi: 10.1128/mSphere.00539-21 (PMC8386399; doi:10.1128/mSphere.00539-21)
Supplement: TABLE S1 [file msphere.00539-21-st001.docx]

| Name | Sequence | Gene |
| --- | --- | --- |
| AfChsA RT Fw | CTGGAGTGTGGCTGGTCTCT | ChsA |
| AfChsA RT Rev | GCGTGTGAAAGCAGTATGGA | ChsA |
| AfTubA RT Fw | TTCCCAACAACATCCAGACC | TubA |
| AfTubA RT Rev | CGACGGAACATAGCAGTGAA | TubA |
| AfGelB RT Fw | CAGGAGGAGAACGACTACGG | GelB |
| AfGelB RT Rev | AGGTCTGGGTTGTGTTGGAG | GelB |
| AfFKS1 RT fw | AAGCAATCGAAGCTCAGGAA | Fks1 |
| AfFKS1 RT Rev | ACCAATCCCATAGAGCGAAC | Fks1 |
| AfGel4 RT Fw | CCTCTGGCCAATGTTGACAA | Gel4 |
| AFGel4 RT Rev | GGTGCGCAGCTGCTTCA | Gel4 |
